# Characterisation of respiratory syncytial virus activity in children and adults presenting with acute respiratory illness at primary care clinics in Singapore, 2014‐2018

**DOI:** 10.1111/irv.12730

**Published:** 2020-02-24

**Authors:** Li Wei Ang, Tze Minn Mak, Lin Cui, Yee Sin Leo, Vernon Jian Ming Lee, Raymond Tzer‐Pin Lin

**Affiliations:** ^1^ National Centre for Infectious Diseases Singapore City Singapore; ^2^ Public Health Group Ministry of Health Singapore City Singapore

**Keywords:** acute respiratory illness, respiratory syncytial virus, virological surveillance

## Abstract

**Background:**

Respiratory syncytial virus (RSV) is an important respiratory pathogen that affects people of all ages.

**Objectives:**

We examined the patterns of RSV circulation in 2014‐2018, and investigated their age‐specific differences in tropical Singapore.

**Methods:**

Nasopharyngeal and/or throat swabs were taken from outpatient attendees for the national influenza virological surveillance among those who presented with acute respiratory illness in the community. Specimens tested negative for influenza were then tested for RSV and other respiratory pathogens.

**Results:**

Among 8436 influenza‐negative specimens tested during the five‐year period, 5.8% (95% confidence interval 5.3%‐6.3%) were positive for RSV. The peak of RSV activity occurred around middle of the year. The age‐specific proportion of RSV detections showed a reverse J‐shaped pattern; RSV positivity was the highest in young children ≤2 years of age (10.9%), followed by those aged 3‐5 years (6.4%) and persons aged ≥65 years (5.3%), while the nadir was observed in the age group of 15‐24 years (1.2%). RSV type A was predominantly circulating in children ≤5 years of age from 2014 to 2015 and 2017, whereas in 2016, they were more affected by type B.

**Conclusion:**

Respiratory syncytial virus was more frequently detected among the two age groups that have been recommended for influenza vaccination; persons ≥65 years of age and children 6 months to <5 years of age. Characterisation of RSV activity in the community helps to better inform public health policies for effective prevention and control interventions.

## 1 INTRODUCTION

Respiratory syncytial virus (RSV), a member of the *Paramyxoviridae* family, is recognised as an important respiratory pathogen that affects people of all ages. Lower respiratory tract infection (LRTI) due to RSV is a leading cause of paediatric hospitalisations worldwide.^1^, ^2^, ^3^, ^4^ There is also substantial disease burden from RSV‐associated acute respiratory illness (ARI) among persons ≥65 years of age.^5^

Most studies that have attempted to quantify the burden of LRTIs were based on hospital‐based surveillance, and they focused on children <5 years of age or subpopulations such as immunocompromised children and adults.^6^ The most common causes of respiratory viral infections are RSV and rhinoviruses in these studies, including those in Asia.^7^, ^8^, ^9^, ^10^ Active surveillance of healthy children aged 6 months to 10 years with influenza‐like illness (ILI) enrolled in a randomised trial at 17 centres in eight countries (including Singapore) between February 2010 and August 2011 demonstrated considerable burden of RSV‐associated illness in the community.^11^

Despite the considerable burden of respiratory viral infections, there are few effective pharmacologic interventions to mitigate the health impact of these pathogens other than for influenza.^12^ In 2016, the World Health Organisation (WHO) piloted a two‐year project to test the feasibility of implementing RSV surveillance based on the Global Influenza Surveillance and Response System (GISRS) in 14 countries across all six WHO regions.^13^ The initiative arose from recognition of the need to provide the evidence base such as seasonality, disease burden and risk groups, to guide future RSV vaccination and other prevention programmes.^13^ As of end 2019, none of the RSV vaccine candidates have reached licensing, and treatment of RSV infection is primarily supportive.

While surveillance of influenza and other respiratory viruses may include testing for RSV, there has been limited data on the epidemiology of RSV, particularly in tropical settings. The aim of this study was to describe the pattern of RSV circulating in the community based on sentinel surveillance of outpatient attendees who presented with ARI in Singapore, a globally connected city‐state in the tropics in Southeast Asia. We further sought to investigate the age‐specific differences in RSV activity.

## 2 METHODS

### 2.1 Virological surveillance and laboratory methods

The Ministry of Health (MOH) conducts the National Surveillance Programme for Influenza (NSPI) throughout the year. For the virological surveillance in the community, nasopharyngeal and/or throat swabs are taken from outpatient attendees with an ARI and measured fever of ≥38°C and cough at government‐funded primary care clinics and sentinel private general practitioner (GP) clinics after obtaining verbal informed consent.^14^, ^15^

The decision to test is based on clinical judgement, and outpatient attendees are not systematically enrolled based on symptoms. For consenting outpatient attendees who fulfil the inclusion criteria, information such as the date of symptom onset, travel history in the past 10 days and influenza vaccination in the past 6 months, if any, are recorded on a data collection form. The specimens are stored in viral transport medium and despatched by courier delivery to the National Public Health Laboratory (NPHL), Singapore, on the same day for nasopharyngeal and/or throat swabs taken on weekdays, and on the following Monday for those taken on weekends.

Testing of the specimens collected for the virological surveillance under NSPI was conducted by NPHL. Real‐time reverse transcription polymerase chain reaction was used to determine influenza virus types and subtypes.^14^, ^15^ All influenza‐negative specimens were then tested for RSV and other respiratory pathogens using available commercial syndromic panels. Prior to September 2014, samples were evaluated using the Seeplex^®^ RV12 ACE Detection kit (Seegene Inc). This was subsequently replaced by the Seeplex^®^ RV15 ACE Detection kit (Seegene Inc), which was used until September 2017. From October 2017 onwards, specimens were evaluated using the FilmArray^®^ Respiratory Panel (FARP) on the FilmArray 2.0 system (BioFire Diagnostics LLC). All assays were performed according to manufacturers’ instruction, and the three kits permit the detection of RSV. Studies have shown that the performance of the FARP for the detection of respiratory viruses was comparable with that of the Seeplex^®^ RV15.^16^, ^17^ Unlike Seeplex^®^ RV12 and RV15, the FARP does not distinguish between RSV types A and B.

### 2.2 Statistical analysis

Seven age groups were considered in our study: ≤2 years, 3‐5 years, 6‐14 years, 15‐24 years, 25‐44 years, 45‐64 years and ≥65 years. In view of the smaller numbers in the age groups of 6‐14 years and ≥65 years, we did stratification by four broad age groups for the analysis on monthly RSV detections and distribution of RSV types among RSV‐positive specimens: ≤5 years, 6‐24 years, 25‐44 years, ≥45 years. We determined the proportion of influenza‐negative specimens tested positive for RSV as a measure of RSV activity in the community. The 95% confidence intervals (CI) for binomial proportions were calculated using Wilson's method. The Mantel‐Haenszel linear‐by‐linear association chi‐square test was used to evaluate whether there was linear trend in annual proportion of RSV detections across the five‐year study period. A two‐proportion *z* test was used to assess the difference in the proportions of RSV‐positive specimens between any 2 years. A RSV type was deemed to be predominant if its proportion was 10% points or higher than that of the other type among RSV‐positive samples. All statistical tests were two‐sided, and *P*‐values <.05 were considered statistically significant.

## 3 RESULTS

During the five‐year period from 2014 to 2018, 16 877 specimens from outpatients who presented with ARI at outpatient setting were collected and 8436 (49.4%) tested negative for influenza. The annual number of influenza‐negative specimens that were tested for RSV increased from 1281 in 2014 to 1892 in 2018 (Table 1). About 45.7% of the influenza‐negative specimens were from children aged ≤5 years, 32.7% from adults aged 25‐64 years and 5.2% from the age group of ≥65 years. The median age was 7 years (interquartile range 2‐38).

**Table 1:** Annual number of influenza‐negative specimens tested for RSV, number and proportion of RSV detections by age group among outpatient attendees who presented with acute respiratory illness in the community, 2014 to 2018

Age group (y)	No. of RSV detections/No. of specimens tested (%) by year					
	2014	2015	2016	2017	2018	2014‐2018
0‐2	55/384 (14.3%)	42/466 (9.0%)	63/588 (10.7%)	61/604 (10.1%)	62/560 (11.1%)	283/2602 (10.9%)
3‐5	17/210 (8.1%)	18/259 (6.9%)	14/257 (5.4%)	18/246 (7.3%)	13/281 (4.6%)	80/1253 (6.4%)
6‐14	1/97 (1.0%)	5/120 (4.2%)	2/136 (1.5%)	3/118 (2.5%)	1/144 (0.7%)	12/615 (2.0%)
15‐24	3/106 (2.8%)	1/135 (0.7%)	2/148 (1.4%)	2/145 (1.4%)	0/136 (0.0%)	8/670 (1.2%)
25‐44	6/249 (2.4%)	3/308 (1.0%)	6/307 (2.0%)	8/370 (2.2%)	8/383 (2.1%)	31/1617 (1.9%)
45‐64	11/155 (7.1%)	4/195 (2.1%)	11/235 (4.7%)	10/277 (3.6%)	12/281 (4.3%)	48/1143 (4.2%)
≥65	4/67 (6.0%)	1/68 (1.5%)	8/92 (8.7%)	5/105 (4.8%)	5/103 (4.9%)	23/435 (5.3%)
All^a^	97/1281 (7.6%)	74/1579 (4.7%)	107/1813 (5.9%)	108/1871 (5.8%)	101/1892 (5.3%)	487/8436 (5.8%)

^a^ Includes 101 outpatient attendees with unknown age.

Among the 8436 influenza‐negative specimens, 487 (4.8%, 95% CI: 5.3%–6.3%) tested positive for RSV. There was no significant linear trend in the annual proportion of RSV‐positive samples across the study period (*P* = .122), and in the seven age groups (all *P* > .10). The proportion of RSV detections dropped significantly from 7.6% in 2014 to 4.7% in 2015 (*P* = .001) (Table 1). The highest RSV positivity was observed in five of the seven age groups in 2014: ≤2 years, 3‐5 years, 15‐24 years, 25‐44 years and 45‐64 years. The nadir of RSV detections occurred in four age groups in 2015: ≤2 years, 25‐44 years, 45‐64 years and ≥65 years.

The RSV positivity was consistently highest in infants and toddlers aged ≤2 years over the five‐year study period (Table 1). The lowest annual RSV positivity was observed in children of school‐going age of 6‐14 years in 2014, and in adolescents and young adults aged 15‐24 years in the ensuing four years.

**Figure 1:**
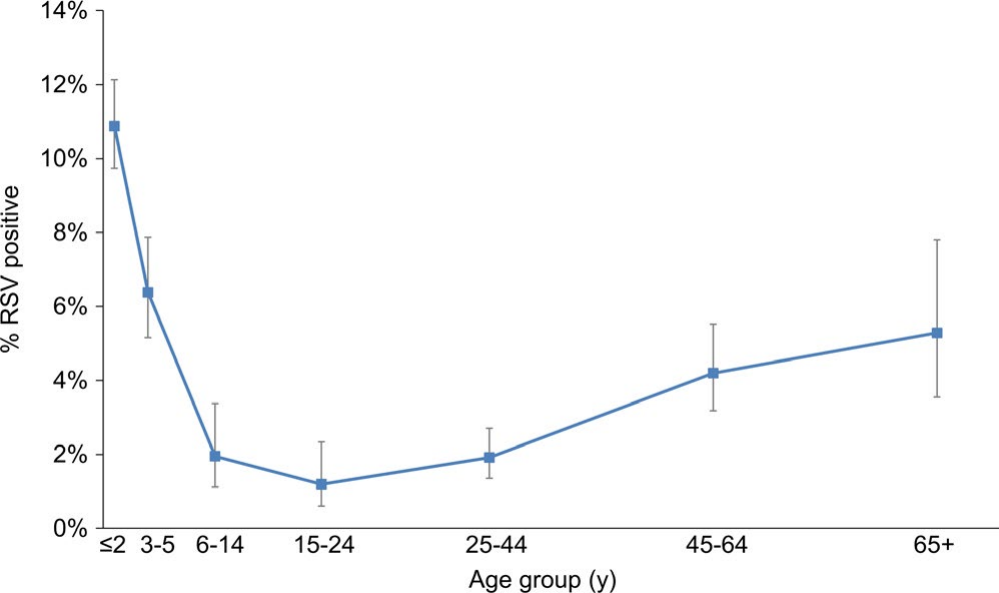
Age‐specific proportion (%) of RSV detections in influenza‐negative specimens of outpatient attendees who presented with acute respiratory illness in the community, 2014 to 2018

The age‐specific proportion of RSV detections showed a reverse J‐shaped pattern over the five‐year study period (Figure 1). The RSV positivity was highest in the age group of ≤2 years at 10.9% (95% CI: 9.7%–12.1%), and lowest in the age group of 15‐24 years at 1.2% (95% CI: 0.6%–2.3%). The second highest proportion of RSV detections was in children aged 3‐5 years (6.4%, 95% CI: 5.2%–7.9%), followed by persons aged ≥65 years (5.3%, 95% CI: 3.5%–7.8%).

**Figure 2:**
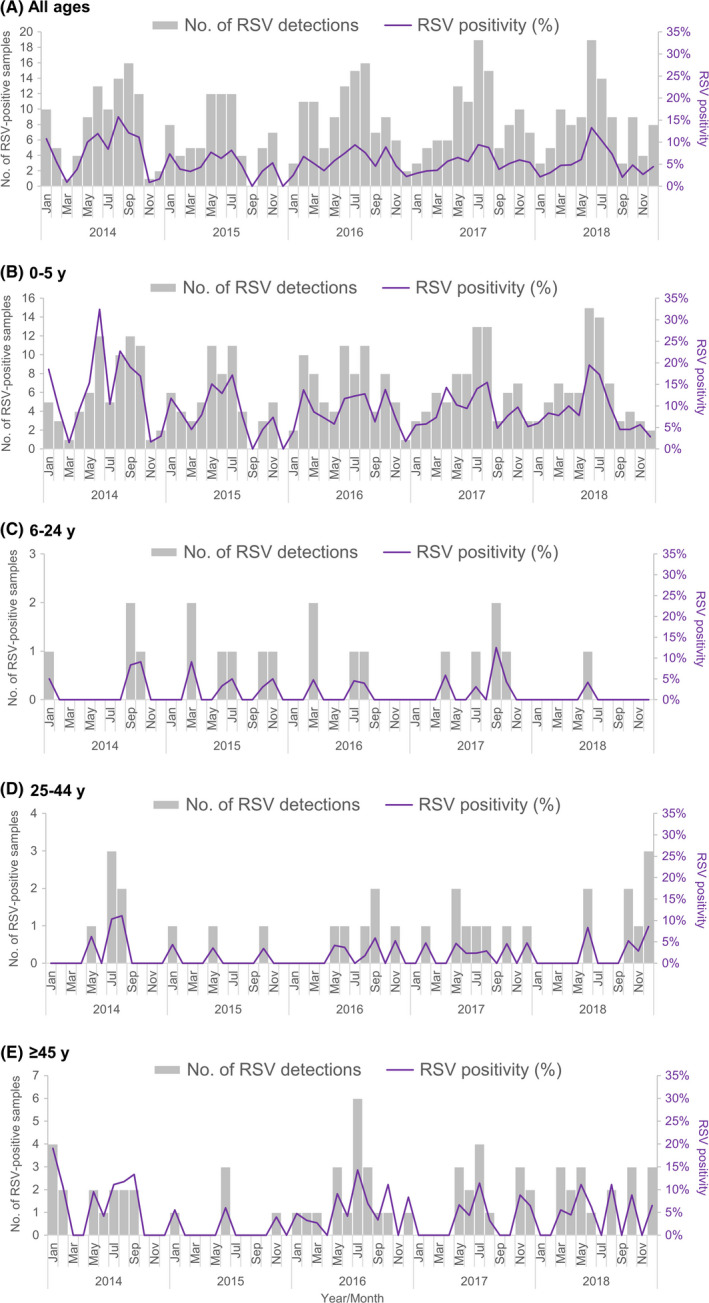
Monthly number and proportion (%) of RSV detections in influenza‐negative specimens of outpatient attendees who presented with acute respiratory illness in the community, 2014 to 2018. A, All ages. B, 0‐5 y. C, 6‐24 y. D, 25‐44 y. E, ≥45 y

The highest RSV positivity was observed in August 2014 across the 60‐month period (Table 2 and Figure 2). There were 3 spikes in monthly RSV detections in each year from 2014 to 2016, whereas the following 2 years each saw a unimodal peak. The peak in monthly proportion tested positive for RSV occurred mostly around middle of the year during the study period; July for each of the 3 years from 2015 to 2017 and June in 2018. Among children aged ≤5 years and adults aged ≥45 years, the highest proportions of RSV detections were observed around middle of the year (Table 2 and Figure 2).

**Table 2:** Highest monthly proportion (%) of RSV detections in influenza‐negative specimens of outpatient attendees who presented with acute respiratory illness in the community by age group, 2014 to 2018

Age group (y)	Highest RSV positivity (no. tested, corresponding period) by year				
	2014	2015	2016	2017	2018
0‐5	32.4% (n = 37, Jun)	17.2% (n = 64, Jul)	13.8% (n = 58, Oct)	15.5% (n = 84, Aug)	19.5% (n = 77, Jun)
6‐24	9.1% (n = 11, Oct)	9.1% (n = 22, Mar)	4.8% (n = 42, Mar)	12.5% (n = 16, Sep)	4.2% (n = 24, Jun)
25‐44	11.1% (n = 18, Aug)	4.3% (n = 23, Jan)	5.9% (n = 34, Sep)	4.8% (n = 21, Feb)	8.6% (n = 35, Dec)
≥45	19.1% (n = 21, Jan)	6.0% (n = 50, Jun)	14.3% (n = 42, Jul)	11.4% (n = 35, Jul)	11.1% (n = 27, May; n = 18, Aug)
All	15.7% (n = 89, Aug)	8.2% (n = 147, Jul)	9.4% (n = 160, Jul)	9.4% (n = 202, Jul)	13.3% (n = 143, Jun)

**Figure 3:**
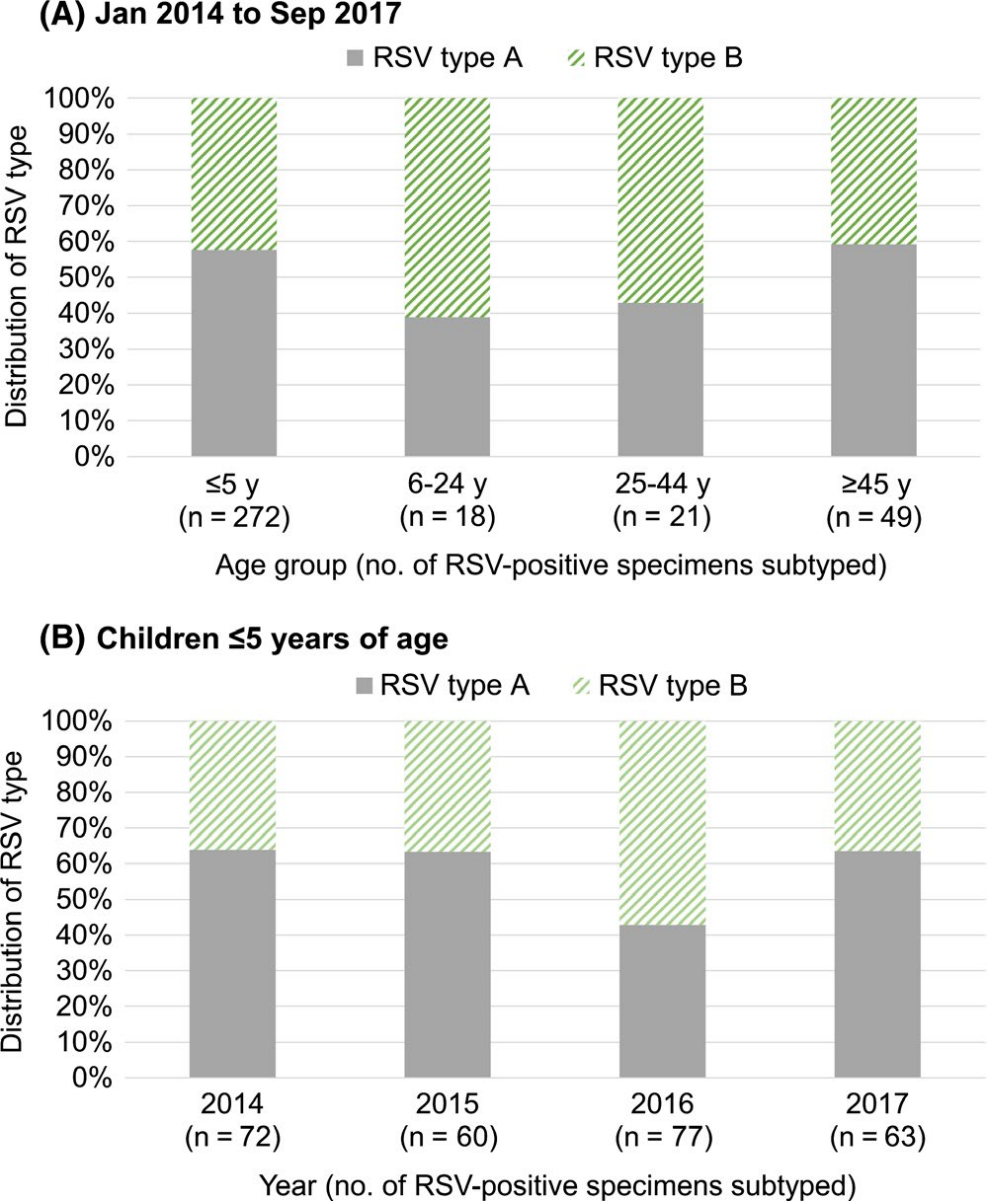
Distribution (%) of RSV type A and B in RSV‐positive specimens (tested negative for influenza) of outpatient attendees who presented with acute respiratory illness in the community by (A) age group for samples subtyped from 2014 to 2017, (B) year of sample collection for children ≤5 y of age

From January 2014 to September 2017 when RSV typing was carried out, the annual number of RSV‐positive samples with typing results ranged from 74 to 107. Children aged ≤5 years and adults aged ≥45 years were more affected by RSV type A, whereas a higher proportion of RSV type B was detected in the age groups of 6‐24 years and 25‐44 years (Figure 3A). Children ≤5 years of age comprised three‐quarters (75.6%) of the RSV‐positive samples which were subtyped, and they were more affected by RSV type A with the exception of year 2016 when RSV type B was predominantly circulating (Figure 3B). The number of RSV‐positive samples in the older age groups was too small to allow for further breakdown by year.

## 4 DISCUSSION

This study examined the patterns of RSV circulation in the local community using specimens tested negative for influenza from the national influenza virological surveillance. RSV was more frequently detected among the two age groups that have been recommended for influenza vaccination; children aged 6 months to <5 years and persons ≥65 years of age. Both seasonal influenza and RSV cause a substantial burden of LRTIs worldwide.^1^, ^2^, ^5^, ^18^ Similar to influenza, RSV can precipitate or exacerbate cardiopulmonary complications.^19^, ^20^, ^21^ It is difficult to differentiate clinically between illnesses caused by influenza and RSV among individuals who present with ILI.^22^ There is a need to estimate the disease burden attributable to each respiratory pathogen for optimal clinical management, especially in the tropics where there are no distinct influenza‐predominant seasons.^22^ Previous studies have characterised influenza activity in outpatient settings and investigated age‐specific differences in distribution of influenza virus (sub)types in Singapore.^14^, ^15^

Based on the national virological surveillance in Singapore, seasonal influenza epidemics usually occur around the start or end and middle of the year.^15^ Our study revealed that the peak of RSV activity among outpatient attendees in the community occurred mostly around middle of the year as well, which coincided with that of influenza. The monthly proportion of RSV detections exceeded the threshold of 10% in two years, 2014 and 2018, which was set by the US Centers for Disease Control and Prevention to define a RSV season.^23^ In temperate climates, the timing of epidemics associated with influenza often overlap with that of RSV.^24^, ^25^ Unlike temperate regions where RSV exhibits distinct seasonality with onset starting in late fall or early winter and ending in late spring,^26^ the epidemiological features of influenza and RSV in the tropics are more varied. We observed three spikes in the monthly RSV positivity in each year from 2014 to 2016, followed by a unimodal peak in the ensuing two years (Figure 2).

The age‐specific RSV positivity in Singapore depicted a reverse J‐shaped pattern; the RSV positivity was highest in infants and toddlers ≤2 years of age, followed by children aged 3‐5 years and persons aged ≥65 years (Figure 1). RSV is a common infection in infancy; it has been estimated that over 95% of children have been infected with RSV by 2 years of age.^27^, ^28^ A prospective study conducted during the peak of five influenza seasons in Canada revealed a higher burden and severity of infections due to RSV compared with influenza in hospitalised children <2 years of age.^29^ While RSV has been primarily seen as a cause of illness in infants and children, studies in hospitalised adults have led to increasing recognition of the RSV‐associated burden among elderly persons.^30^, ^31^, ^32^ Future studies should explore the impact of RSV among persons aged ≥65 years.

Consistent with what has been reported in the literature, children aged ≤5 years had significantly higher proportion of RSV detections (9.4%, 95% CI: 8.5%‐10.4%) than older age groups. The magnitude of RSV detections in influenza‐negative specimens of outpatient attendees presenting with ARI in our study was similar to that of a study in Hong Kong with a subtropical climate^33^; a significantly higher proportion of RSV was also detected among young children ≤5 years of age (10.9%) who presented with ARI in a community outpatient setting from February 2007 to December 2010, compared with the age group of 6‐15 years (1.0%). About 10% of the specimens from paediatric inpatients at two government acute‐care hospitals tested positive for RSV in 2014‐2018 (unpublished data). These two hospitals with paediatric departments covered about two‐thirds of hospitalisations for children <15 years of age in Singapore.^34^

While RSV predominately affect young children,^35^ there has been more evidence indicating that the impact on older adults, particularly the elderly, is similar to that of seasonal influenza.^20^, ^36^, ^37^ In Hong Kong, RSV patients were more likely to have underlying chronic lung disease and major systemic co‐morbidities when compared with influenza patients of similar age, while the mortality rate of RSV patients with severe lower respiratory complications was similar to that of seasonal influenza.^38^ In the United States, RSV‐attributable healthcare resource use (hospital stays, emergency room/urgent care visits, ambulatory visits and outpatient visits) and costs across age groups were substantial, with the highest burden in the elderly aged ≥65 years.^39^

In our study, RSV type A was predominantly circulating in children ≤5 years of age from 2014 to 2015 and 2017, whereas in 2016, they were more affected by type B (Figure 3B). The antigenic differences between the two RSV types have been found to affect susceptibility to infection or disease.^40^ However, differences in the clinical severity between the two RSV types remain unclear. In a prospective study on infants hospitalised with bronchiolitis, RSV type A infection resulted in greater severity,^41^ which was in agreement with most published studies.^42^, ^43^, ^44^ Other studies had either found infection due to RSV type B to be more severe,^45^ or no significant association between clinical severity of RSV infection and type.^46^, ^47^ Possible differences in disease severity between RSV types A and B might have important implications for prevention and treatment strategies, including future vaccination and clinical management.^43^

As the RSV activity in this study was reflected by the proportion of RSV‐positive specimens that had been tested negative for influenza, we could not determine the frequency of RSV and influenza co‐infections during the five‐year period. Since June 2019, NPHL has started testing all specimens collected under the national influenza virological surveillance against a panel of respiratory pathogens including RSV. During the three‐month period from June to August 2019, RSV was detected in 1.4% of the influenza‐positive samples (unpublished data). Hence, the proportion with co‐infection of influenza and RSV in the community is likely to be small. A study on respiratory viral infections based on community specimens of children ≤15 years of age in Hong Kong revealed that the frequency of co‐detections with RSV was very low at 1.0%, and RSV was more frequently co‐detected in children ≤5 years of age than in the older age group of 6‐15 years (2.3% vs 0.1%).^33^ In the United States, a retrospective cross‐sectional study of children evaluated for viral respiratory infection between July 2010 and June 2013 found that the observed incidence of co‐infections of RSV and influenza was significantly less than the expected incidence even when both viruses were co‐circulating.^48^ In a study on circulation of respiratory viruses from 2002 to 2017 in Victoria, Australia, co‐detections of other respiratory viruses with influenza A and B infections were also uncommon in positive samples (6%), and detection of co‐infections was less likely with older age.^49^

The data for our study originated from a nationwide network of primary care outpatient clinics in public and private sectors for the influenza virological surveillance. In Singapore, there are 20 government primary care clinics and about 1700 private GP clinics as of 2018, with private GP clinics meeting about 80% of the total primary care demand.^50^ ARI was the top medical condition seen at government primary care clinics and private GP clinics in the Primary Care Survey conducted by MOH, and it constituted 20% of all diagnoses in 2014.^51^ There are about 30 active private GPs and 20 government primary care clinics representing all regions in Singapore in the virological surveillance programme for influenza. Hence, the findings can be deemed to be a good representation of the general patterns of virus circulation in the community.

There are some limitations to this study. RSV detection was confined to specimens which had been tested negative for influenza during the study period, as the primary objective of the virological surveillance programme is to monitor influenza activity in outpatient attendees presenting with ARI/ILI in the community. As primary care practitioners may have obtained swabs from outpatient attendees who presented with ARI based on judgemental sampling (clinical suspicion of having a positive test), we could not rule out the possibility of selection bias and surveillance artefacts. Health‐seeking behaviour and willingness to provide consent for testing might differ across the age groups. However, we did not have information on the number of outpatients who presented with an ARI and measured fever of ≥38°C and cough at government‐funded primary care clinics and sentinel private GP clinics to estimate the proportion enrolled for testing.

In conclusion, RSV was most frequently detected among age groups that have been recommended for influenza vaccination; young children aged ≤5 years followed by persons aged ≥65 years who presented with ARI in the local outpatient setting. Given the varied patterns of RSV circulation shown in this study, there is a need for continuous surveillance throughout the year. The characterisation of RSV activity provides insights into the epidemiological aspects for review of public health strategies to prevent and reduce transmission of RSV in the community.
